# Supplementary material for: EFE-Mediated Ethylene Synthesis Is the Major Pathway in the Citrus Postharvest Pathogen Penicillium digitatum during Fruit Infection
Source: J Fungi (Basel). 2020 Sep 17;6(3):175. doi: 10.3390/jof6030175 (PMC7558865; doi:10.3390/jof6030175)
Supplement: Supplementary file 1 [file jof-06-00175-s001.zip › jof-909456_supplementarymaterial_vfinal.docx]

EFE-mediated ethylene synthesis is the major pathway in the citrus postharvest pathogen *Penicillium digitatum* during fruit infection

Ana-Rosa BALLESTER and Luis GONZÁLEZ-CANDELAS

**SUPPLEMENTARY MATERIAL**

Deletion of the P. digitatum efeA gene

To obtain knockout mutants, 1389 and 1547 bp DNA fragments upstream and downstream of the gene of interest, respectively, were amplified and introduced into the plasmid pRFHU2 by the USER-friendly cloning technique [1]. Gene disruption by homologous recombination was conducted using the in-house constructed pRFHU-PdefeA plasmid, which contains the hygromycin resistant cassette as a positive selection marker (Figure S1). Knockout mutants were confirmed by PCR with specific primers for the amplification of the region that flanked both the 5’ and 3’ end of construction in combination with the primers located in the hygromycin resistance marker, and for the hygromycin resistance gene (Figure S1A-B, and Table S1). Monosporic isolates were obtained from these transformants and were further validated by PCR using the primers located in the *efeA* coding region. Two knockout mutants (Δ1f and Δ23c) and one ectopic transformant were selected for the determination of the number of T-DNA copies inserted into the genome by quantitative PCR using the *P. digitatum* strain Pd1 as a control and the β-tubulin encoding gene as a reference. The two knockout and ectopic mutants contained one single copy of the T-DNA integrated into the genome (Table S2).

**Table S1**. Primers employed in the study. The 5’ deoxyuridine extension parts in the primers employed for the assembly of the USER cloning sites are underlined.

| Primer name | Sequence (5’→3’) | Used for/Position: |
| --- | --- | --- |
| PDIP_08660_O1 | GGTCTTAAUACGTCGAGTGGACTGGGATA | Construction of knockout mutant |
| PDIP_08660_O2 | GGCATTAAUAAGCTTTTTGTCTGCTGGCG | Construction of knockout mutant |
| PDIP_08660_A3 | GGACTTAAUTGTCACATTCACTGCCGTCT | Construction of knockout mutant |
| PDIP_08660_A4 | GGGTTTAAUATGCATCACCACCTTGGGAG | Construction of knockout mutant |
| RF1 | AAATTTTGTGCTCACCGCCTGGAC | *E. coli* transformants |
| RF2 | TCTCCTTGCATGCACCATTCCTTG | *E. coli* transformants |
| RF5 | GTTTGCAGGGCCATAGAC | *E. coli* transformants |
| RF6 | ACGCCAGGGTTTTCCCAGTC | *E. coli* transformants |
| PDIP_08660_1F | ACGACACAAAAGGGCAGGAT | 5’ upstream of O1 |
| PDIP_08660_2R | TCTTTGATCTCAGCGCCTCC | 3’ downstream of A4 |
| PDIP_08660_3F | TGCCTCCGAATCAAAAGGCT | Gene of interest-RGE |
| PDIP_08660_4R | CACCTCATCCTGAGCTGCAA | Gene of interest-RGE |
| HPHTer2 | GCTCCGTAACACCCAATAC | Hygromycin gene |
| HPHPro4 | GCACCAAGCAGCAGATGATA | Hygromycin gene |
| HMBF1 | CTGTCGAGAAGTTTCTGATCG | Hygromycin gene |
| HMBR1 | CTGATAGAGTTGGTCAAGACC | Hygromycin gene |
| PDIP_08660_5F | CATCCACCCCTCACAAGGTC | T-DNA copy number |
| PDIP_08660_6R | CGTAAAGCTTCGCAACTGGG | T-DNA copy number |
| betatubPDIG1 | CGATGGCGATGGACAGTAAGTTT | T-DNA copy number |
| betatubPDIG2 | TTGGTTCGTGGTCGTTGTACTCA | T-DNA copy number |
|  |  |  |

**Table S2**. Determination of the T-DNA copy number integrated into the genome of mutants based on the formula described by Pfaffl [2].

| Sample | Cq (*efeA*) | Cq (*tub*) | T-DNA copy number |
| --- | --- | --- | --- |
| Pd1 | 21.22 ± 0.10 | 21.89 ± 0.25 | - |
| ect | 19.83 ± 0.05 | 21.39 ± 0.07 | 0.86 |
| Δ1f | 20.61 ± 0.11 | 21.27 ± 0.00 | 1.00 |
| Δ23c | 21.29 ± 0.02 | 21.98 ± 0.01 | 1.02 |

**Table S4**. Ethylene production by the different strains grown on PDA plates and incubated at 24ºC for up to 14 days. Ethylene production is expressed as nL/h per plate. The values represent the mean of three biological replicates. The values labeled with the same letter at each time point do not differ at the 95% confidence level based on the Tukey's honestly significant difference (HSD) procedure.

| Days | Pd1 | Ectopic | Δ1f | Δ23c |
| --- | --- | --- | --- | --- |
| 1 | 0.7^a^ | 1.6^a^ | 0^a^ | 2.2^a^ |
| 2 | 0 | 0 | 0 | 0 |
| 3 | 2.0^a^ | 0.7^a^ | 0^a^ | 0^a^ |
| 4 | 17.9^a^ | 16.4^a^ | 0^b^ | 0^b^ |
| 5 | 75.3^b^ | 167.1^a^ | 0^c^ | 0^c^ |
| 6 | 391.6^a^ | 543.1^a^ | 0^b^ | 0^b^ |
| 7 | 520.3^a^ | 638.9^a^ | 0^b^ | 0^b^ |
| 8 | 628.3^a^ | 551.4^a^ | 0^b^ | 0^b^ |
| 11 | 490.3^a^ | 349.9^b^ | 0^c^ | 0^c^ |
| 14 | 233.2^a^ | 110.3^b^ | 0^c^ | 0^c^ |

**Table S5**. Ethylene production, expressed as nL/g h, during the growth of parental strain Pd1 and the Δ1f knockout mutant on inactivated orange peel discs. Orange peel discs were inoculated with a conidia suspension (10^6^ conidia/mL) of both strains in 15 mL glass tubes at 24°C for up to 21 days post-inoculation (dpi). Values are the mean of five biological replicates±SEM. As values in the Δ1f mutant were almost always 0, except in five tubes of the 60 tubes analyzed for this mutant, a variance analysis cannot be performed and, thus, no statistical test can be conducted.

| Days | Pd1 | Δ1f |
| --- | --- | --- |
| 1 | 0 ± 0 | 0 ± 0 |
| 2 | 0 ± 0 | 0 ± 0 |
| 3 | 0.8 ± 0.3 | 0 ± 0 |
| 4 | 11.5 ± 3.1 | 0 ± 0 |
| 5 | 33.6 ± 3.2 | 0 ± 0 |
| 6 | 44.1 ± 7.8 | 0 ± 0 |
| 7 | 88.5 ± 12.7 | 0 ± 0 |
| 8 | 175.7 ± 20.6 | 0 ± 0 |
| 9 | 245.0 ± 22.7 | 0.3 ± 0.3 |
| 11 | 279.2 ± 16.0 | 0 ± 0 |
| 14 | 182.2 ± 10.0 | 0 ± 0 |
| 21 | 37.8 ± 6.5 | 0 ± 0 |


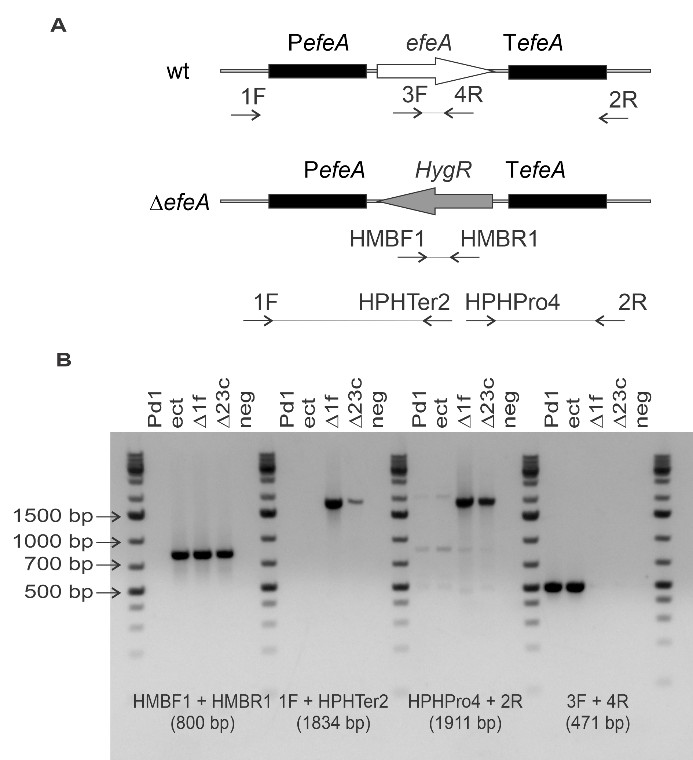


**Figure S1**. Analysis of *Penicillium digitatum efeA* transformants. (**a**) Diagram of the wild-type locus and the *efeA* replacement with the Hyg^R^ selectable marker from the pRFHU-PdefeA plasmid by homologous recombination to generate the Δ*efeA* mutants. The primers used for the analysis of the transformants are shown (Table 1). (**B**) The polymerase chain reaction (PCR) analysis of the wild-type Pd1 strain, an ectopic mutant (denoted as “ect”) and two knockout mutants (Δ1f and Δ23c).

1. Frandsen, R.J.; Andersson, J.A.; Kristensen, M.B.; Giese, H. Efficient four fragment cloning for the construction of vectors for targeted gene replacement in filamentous fungi. BMC Mol. Biol. **2008**, 9, 70, doi:10.1186/1471-2199-9-70.

2. Pfaffl, M.W. A new mathematical model for relative quantification in real-time RT–PCR. Nucleic Acids Res. **2001**, 29, e45, doi:10.1093/nar/29.9.e45.

| 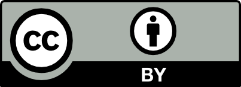 | © 2020 by the authors. Submitted for possible open access publication under the terms and conditions of the Creative Commons Attribution (CC BY) license (http://creativecommons.org/licenses/by/4.0/). |
| --- | --- |
